# Hypereosinophilic syndrome onset with Loeffler's endocarditis after COVID-19 infection^[Author-notes jeac150-FM1]^

**DOI:** 10.1093/ehjci/jeac150

**Published:** 2022-07-28

**Authors:** Massimo Mapelli, Claudia Cefalù, Denise Zaffalon, Andrea Annoni, Piergiuseppe Agostoni

**Affiliations:** Centro Cardiologico Monzino, IRCCS, Milan, Italy; Department of Clinical Sciences and Community Health, Cardiovascular Section, University of Milan, 20122 Milan, Italy; Centro Cardiologico Monzino, IRCCS, Milan, Italy; Department of Cardiothoracovascular, Azienda Sanitaria Universitaria Giuliano Isontina (ASUGI), University of Trieste, Trieste, Italy; Centro Cardiologico Monzino, IRCCS, Milan, Italy; Centro Cardiologico Monzino, IRCCS, Milan, Italy; Department of Clinical Sciences and Community Health, Cardiovascular Section, University of Milan, 20122 Milan, Italy

A 37-year-old, known to have allergic asthma and with normal blood count performed 3 months earlier, was admitted during the COVID-19 outbreak (February 2022) due to new-onset chest discomfort, dyspnoea, and mild fever. Physical examination revealed dark-red, linear lesions in the nail beds consistent with splinter haemorrhages and erythematous papular lesions on the palms and soles consistent with Janeway lesions (*Panel A*). He reported a recent uncomplicated COVID-19 infection that required no therapy (first COVID-19 positive swab 21 days before admission). At admission, he was mild febrile (37.5°C), tachycardic with no signs of heart failure. Electrocardiography showed sinus rhythm with negative T waves V2–V6 (*Panel B*). Laboratory tests revealed leucocytosis (16.6×10^3^/μL) with 53.5% eosinophils (8.9×10^3^/μL). High-sensitive troponin I was high (160.5 ng/L) showing progressive reduction throughout hospitalization. Echocardiography showed severe left ventricle apical endomyocardial thickening, apical thrombus, and left ventricular hypertrophy compatible with a Loeffler’s syndrome (*Panel C*). To confirm the diagnosis, a cardiac magnetic resonance was performed corroborating these findings (*Panel D*) and excluding associated myocardial oedema (normal T2-weighted short-tau inversion recovery images). To exclude associated coronary artery disease or eosinophil-related lung involvement, a chest computed tomography scan was performed showing normal aspect of lung parenchyma and coronary arteries. The patient was treated with corticosteroids and subcutaneous heparin, and rapid clinical picture and eosinophil values normalization was observed (*Panel D*). At 3-month follow-up, he is asymptomatic, and the echocardiogram demonstrated almost complete thrombosis resolution. Loeffler's endocarditis is a cardiac manifestation of hypereosinophilia leading to diastolic dysfunction, valvulopathy, or—as in this case—embolic events. COVID-19 infection, although paucisymptomatic, may have functioned as a trigger for developing a previously completely silent eosinophilic syndrome. Promptly recognizing signs and symptoms allows the correct treatment to be initiated, usually ensuring rapid improvement.

**Figure:**
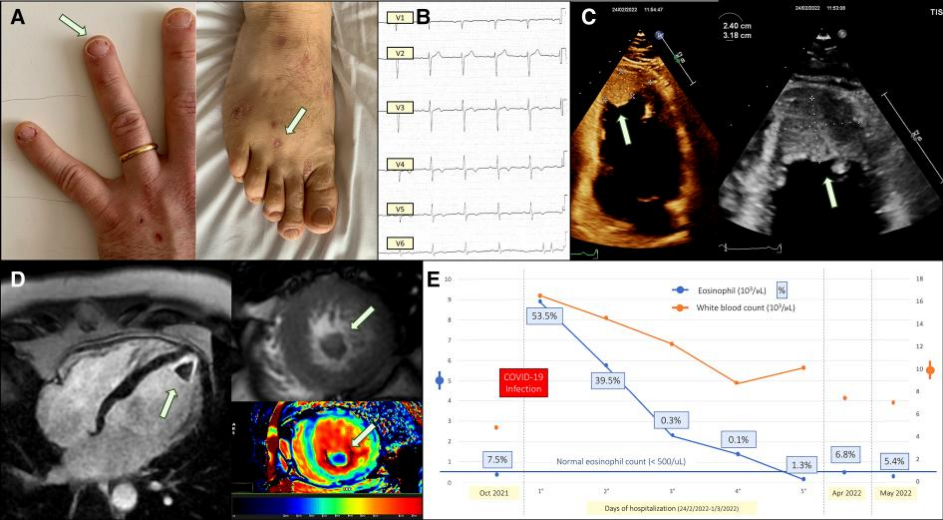


**Funding**: This research was supported by the Italian Ministry of Health-Ricerca Corrente to Centro Cardiologico Monzino IRCCS.

